# Encapsulated Ferritin-like Proteins: A Structural Perspective

**DOI:** 10.3390/biom14060624

**Published:** 2024-05-25

**Authors:** Elif Eren, Norman R. Watts, Felipe Montecinos, Paul T. Wingfield

**Affiliations:** Protein Expression Laboratory, National Institute of Arthritis and Musculoskeletal and Skin Diseases, National Institutes of Health, Bethesda, MD 20892, USA

**Keywords:** metal homeostasis, encapsulin, ferritin-like protein, iron, bacterial ferritins, ferroxidase, bacterioferritins

## Abstract

Encapsulins are self-assembling nano-compartments that naturally occur in bacteria and archaea. These nano-compartments encapsulate cargo proteins that bind to the shell’s interior through specific recognition sequences and perform various metabolic processes. Encapsulation enables organisms to perform chemical reactions without exposing the rest of the cell to potentially harmful substances while shielding cargo molecules from degradation and other adverse effects of the surrounding environment. One particular type of cargo protein, the ferritin-like protein (FLP), is the focus of this review. Encapsulated FLPs are members of the ferritin-like protein superfamily, and they play a crucial role in converting ferrous iron (Fe^+2^) to ferric iron (Fe^+3^), which is then stored inside the encapsulin in mineralized form. As such, FLPs regulate iron homeostasis and protect organisms against oxidative stress. Recent studies have demonstrated that FLPs have tremendous potential as biosensors and bioreactors because of their ability to catalyze the oxidation of ferrous iron with high specificity and efficiency. Moreover, they have been investigated as potential targets for therapeutic intervention in cancer drug development and bacterial pathogenesis. Further research will likely lead to new insights and applications for these remarkable proteins in biomedicine and biotechnology.

## 1. Introduction

Earth was formed around 4.6 billion years ago (Ga) [1]. During the following few million years, conditions suitable for life developed when the surface of the Earth rapidly cooled, giving rise to heavy rains that ultimately formed oceans [2]. Life on Earth in the form of anaerobic microscopic organisms likely began an estimated 3.8–3.5 Ga, long before the oxygenation of the atmosphere [3]. Around 4 Ga, the seawater contained high levels of H_2_S, creating a reducing environment with an estimated potential of around −0.2 V [4]. Therefore, elements with higher negative reduction potentials, such as Fe^+2^ and Mg^+2^, were highly available in their free ionic forms and could subsequently be incorporated into the cells [1]. As life evolved, available metals became an essential part of biological systems. Eventually, two important geological events led to the oxygenation of the atmosphere and the oceans: the Great Oxidation Event (GOE), which took place around 2.5–2.0 Ga, and the Neoproterozoic Oxygenation Event (NEO), which happened roughly 2.0 billion years after the GOE [5]. As a result, the prevailing iron chemistry was altered significantly. The predominant form of iron switched from the soluble ferrous (Fe^+2^) form to the insoluble ferric (Fe^+3^) form [6].

Consequently, the availability of oceanic iron changed from 10^−7^ M (Fe^+2^) under prebiotic (anaerobic) conditions to 10^−18^ M under aerobic conditions [4]. Despite the significant decrease in its availability, iron remained an essential co-factor of many enzymatic reactions due to its interconvertible redox states, large redox potential window (−600 to +500 mV), and its ability to adopt different spin states (high or low) formed by both the Fe^+2^ and Fe^+3^ depending on the ligand environment [6]. However, the presence of O_2_ and aerobic respiration presented a new problem, the Fenton reaction [7]. This reaction, described by H. J. Fenton in the late 19th century, is the enhanced oxidative potential of H_2_O_2_ when iron acts as a catalyst [8]:

Fe^+2^ + H_2_O_2_ → Fe^+3^ + OH^•^ + OH^−^

When this reaction occurs in biological systems, reactive hydroxyl radicals can damage proteins, lipids, carbohydrates, and DNA, eventually leading to cell death [9]. Therefore, bacteria developed several mechanisms to circumvent the problems of iron solubility/availability and intracellular iron toxicity.

Similar to other essential metals, cellular iron homeostasis is described by two key concepts: the quota, which is the total iron content of the cell (atoms/cell), and the labile iron pool, which is a subset of the quota and defined as a pool of redox-active iron that can be readily exchanged for cellular iron metabolism [10]. To give an idea, when *Escherichia coli* (*E. coli*) is grown exponentially under anaerobic conditions, the total content of iron/cell is ~1.2 × 10^6^ atoms, but the labile iron pool is ~1% of these atoms (~10^4^ atoms) [11]. Within the bacterial cell, the majority of the cellular iron is bound to proteins, where it is incorporated into Fe-S cluster enzymes, hemeproteins, mononuclear Fe^+2^–dependent enzymes, etc. [10]. On the other hand, in the reducing environment of the cytoplasm, the labile iron pool is primarily formed by Fe^+2^-glutathione (Fe^+2^-GSH) or other low-molecular-weight thiol complexes that buffer the thermodynamically available free Fe^+2^ [10,12].

The intracellular iron concentrations are sensed directly or indirectly by metalloregulator proteins such as the ferric uptake regulator (Fur) or its functional analogs such as IdeR (in actinomycetes) and Irr (in alpha-proteobacteria) [13,14,15,16,17]. These metalloregulators are transcriptional repressors or activators that regulate genes implicated in iron uptake, storage, and efflux [18,19,20,21]. For instance, in *Bacillus subtilis* (*B. subtilis*) under iron limiting conditions, de-repression of numerous Fur-regulated operons allows the increased expression of siderophores and upregulation of Fe^+3^ uptake pathways such as the YfmCDEF ABC transporter involved in citrate-dependent Fe^+3^ import, or the FeuABC transporter and the YusV ATPase for siderophore-dependent Fe^+3^ uptake [10,22]. In addition, Fur upregulates the FsrA-dependent iron-sparing response, in which FsrA functions to repress “low-priority” iron-containing enzymes [23]. In the presence of excess iron, Fur directly induces the expression of the PfeT, a P1B4-type ATPase that effluxes Fe^+2^ [24].

Besides iron sensing, uptake, and efflux mechanisms, iron storage is another widespread mechanism in maintaining bacterial iron homeostasis. In bacteria, the labile iron pool’s primary role is likely to provide enough iron for the metalation of the iron proteome. However, when the concentration of the labile iron pool exceeds the metabolic requirement, the excess is sequestered in a non-reactive state by dedicated iron storage proteins belonging to the ferritin-like superfamily [25,26]. This storage system prevents the potentially catastrophic consequences of the Fenton reaction and remobilizes the iron to satisfy cellular requirements during starvation. The main bacterial iron storage proteins are bacterial ferritins (Ftns), bacterioferritins (Bfrs), DNA–binding proteins from starved cells (Dps), and encapsulated ferritin-like proteins (FLPs) [27,28,29,30,31,32]. This review will focus on the structural and functional characteristics of the encapsulated FLPs.

## 2. Encapsulins

Encapsulins are a class of prokaryotic protein nano-compartments formed by the self-assembly of a shell protein called the protomer that encapsulates cargo proteins. They are involved in several metabolic pathways, such as iron storage in the mineral form, mitigation of oxidative stress, anaerobic ammonium oxidation, catabolism, and sulfur metabolism [33,34,35,36,37]. The functional role of an encapsulin is determined by its cargo protein(s).

**Figure 1:**
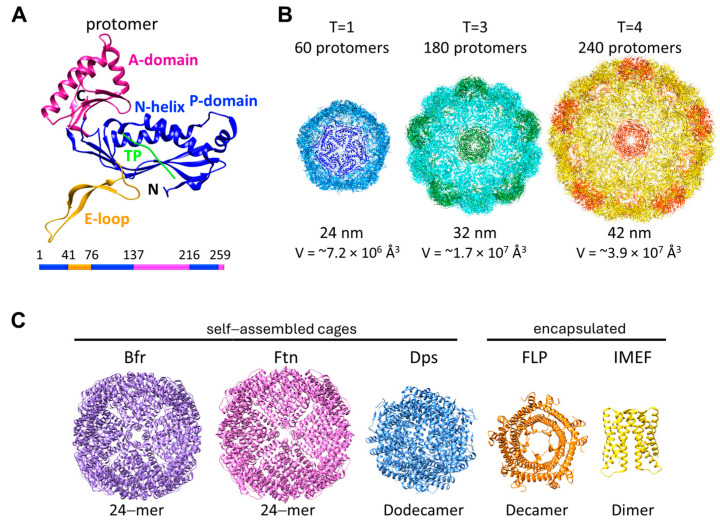
Encapsulins and FLPs. (**A**) Domain organization of the encapsulin protomer (shell protein). A targeting peptide bound to the protomer is shown in green. (**B**) Encapsulins with varying sizes from *H. ochraceum* (T = 1, PDB ID: 7odv), *M. xanthus* (T = 3, PDB ID: 7s20), and *Q. thermotolerans* (T = 4, PDB ID: 6nj8). (**C**) Different types of FLP superfamily proteins. *E. coli* bacterioferritin (Bfr, PDB ID: 3e1m), purple; *E. coli* ferritin (Ftn, PDB ID: 4ztt), pink; *N. punctiforme* DPS (PDB ID: 5hjh), blue; *R. rubrum* EncFtn (PDB ID: 5da5), orange; and *Q. thermotolerans* IMEF (PDB ID: 6n63), gold.

Encapsulin protomers consist of three conserved domains: the axial domain (A-domain), peripheral domain (P-domain), and extended loop (E-loop) (Figure 1A). The protomers possess the bacteriophage HK97-fold, which is widespread among viruses from bacteriophages to herpesviruses [38,39,40,41,42]. The versatility of the HK97-fold enables the formation of viral capsids or icosahedral protein shells with sizes varying from 24 nm (T = 1) to 180 nm (T = 52), composed of 60 to more than 3,000 monomers [43]. The encapsulin shell sizes vary from 20–42 nm with varying triangulation numbers: T = 1 (60-mer), T = 3 (180-mer), or T = 4 (240-mer) (Figure 1B) [35,36,41,44,45,46,47]. The triangulation number is determined by the arrangement of the E-loop between the protomer subunits [48,49]. The cargo proteins are tethered to the inside of the encapsulins via short peptides, called the targeting peptides (TPs), located at the end of their C-termini or less frequently by more extended N-terminal encapsulation-mediating domains [50,51]. The TP binding sites for several encapsulins (T = 1, T = 3, and T = 4) have been determined. These binding sites are located at the P-domain in a hydrophobic cleft formed between the N-terminal helix and the three-stranded-β-sheet (Figure 1A) [41,45,48].

Encapsulins have been classified into four distinct families based on their cargo types and operon organization, and on computational analysis [49,52,53]. The Family 1 encapsulins have been identified in Proteobacteria, Actinobacteria, Firmicutes, and Myxobacteria and are involved in mitigating oxidative stress and iron storage [49,52,53]. These encapsulins vary in size and can encapsulate diverse classes of FLPs [49]. Family 2 encapsulins represent the most numerous encapsulin systems, and the majority are found in Actinobacteria, Proteobacteria, Bacteroidetes, and Cyanobacteria [49]. Family 2 members are most often associated with different types of cargo enzymes, such as desulfurase, poly-prenyl transferase, xylulose kinase, and terpene cyclase [49,54]. Both Family 3 and Family 4 encapsulins have been identified by computational genome mining and are yet to be experimentally characterized [49,54].

## 3. Encapsulated FLPs

Bacterial ferritin-like superfamily proteins, including the Ftn, Bfr, and Dps family members, have a four α-helix bundle structure, and they self-assemble into multi-subunit cage-like structures with interior cavities that act as iron storage reservoirs (Figure 1C) [29]. The Ftn and Bfr family members comprise 24 subunits, which assemble into a cage with octahedral 432-point symmetry [26,27,29]. The quaternary structure of a Bfr is equivalent to that of a Ftn except that there is a heme group between the subunits [29]. The external diameter of these cages is ~12 nm. The diameter of the internal cavity is ~6–8 nm and can accommodate up to 4500 iron atoms in mineralized form [26]. On the other hand, Dps members form 12 subunit cages with tetrahedral 23-point symmetry [26]. These form smaller cages with an external diameter of ~9 nm and an internal cavity diameter of ~5 nm, which can store ~500 iron atoms [26].

**Figure 2:**
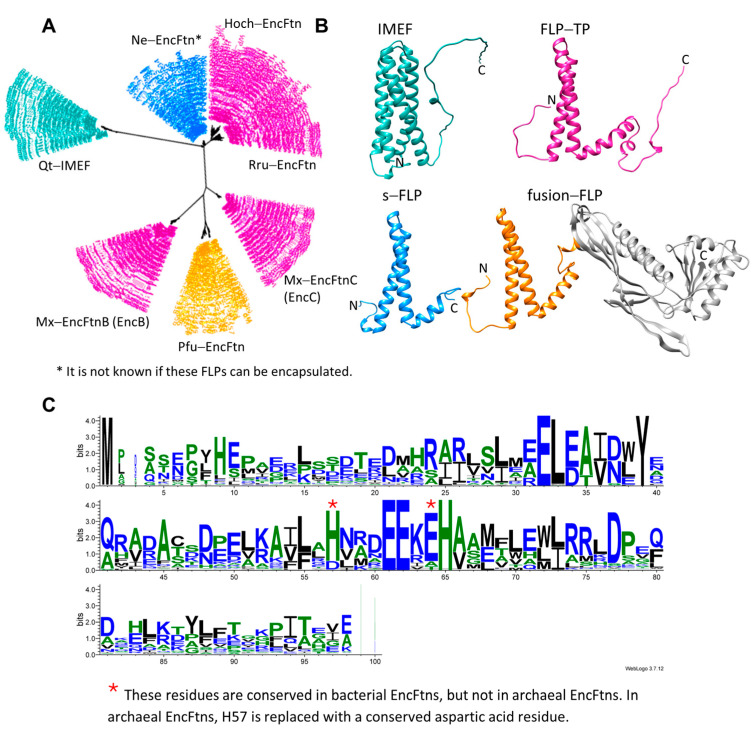
Comparisons of different types of encapsulated FLPs. (**A**) An unrooted phylogenetic tree was obtained by aligning sequences of different types of encapsulated FLPs. For each type of FLP, the 50 closest homolog sequences were obtained from the basic local alignment search tool (BLAST). Sequence alignment was conducted using the COBALT multiple alignment tool. (**B**) Structures of different types of encapsulated FLPs: IMEF (PDB ID: 6n63, aa 143–192 were predicted by AlphaFold), green; FLP-TP (PDB ID: 5da5, aa 1–6 and 98–140 were predicted by AlphaFold), magenta; s-FLP (PDB ID: 3k6c), blue; and fusion–FLP (aa 2–99, PDB ID: 5n5e and aa 111–345 PDB ID: 2e0z; aa 100–110 were predicted by AlphaFold), where the FLP region is shown in orange and the protomer in gray. (**C**) The logo of aligned FLP sequences highlights the conserved residues (created by WebLogo3).

Unlike cytoplasmic FLPs, the encapsulated FLPs do not self-assemble into cages. Instead, the iron is stored inside the encapsulin cage while the FLP carries out the ferroxidase activity. Due to their greater size, encapsulin–FLP systems have a much greater iron storage capacity than Ftns, Bfrs, and Dps proteins. To date, structures of several encapsulated FLPs from archaea and bacteria have been solved by X-ray crystallography (Table 1) [28,45,48,55]. These structures represent four different classes of FLPs: FLPs with a C-terminal targeting peptide (FLP-TP; PDB ID: 5da5, 5n5f, 7s5c, 7s5k, and 7s8t), fusion–FLPs where the FLP is fused to the shell protein (fusion–FLP; PDB ID: 5n5e), shorter FLPs without long disordered C-termini or an apparent C-terminal targeting peptide (s-FLP; PDB ID: 3k6c), and four α-helix bundle FLPs (Iron-Mineralizing Encapsulin-Associated Firmicute, IMEF; PDB ID: 6n63) (Figure 2). FLP-TPs, s-FLPs, and IMEF are predominantly found in bacteria, while fusion–FLPs are commonly found in archaea.

**Figure 3:**
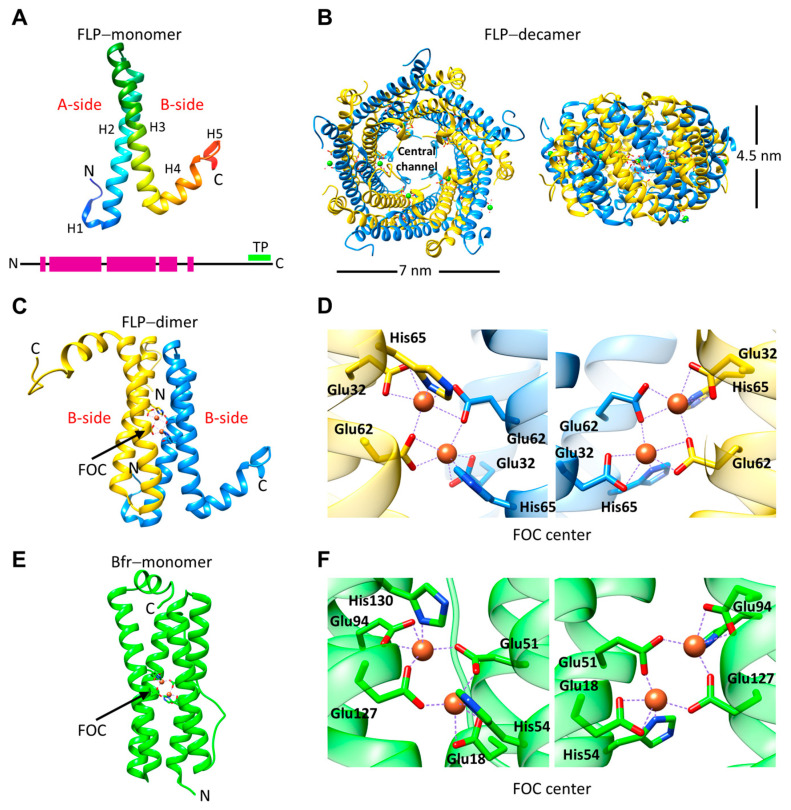
The structure of FLP-TP. (**A**) The Rru-EncFtn monomer (PDB ID: 5da5). The two faces involved in dimer formation are indicated as sides A and B. The disordered C-terminal region is not visible in the crystal structure. The secondary structure is shown as a scheme at the bottom: α-helices are shown in magenta, and the C-terminal TP is indicated with a green line. (**B**) The Rru-EncFtn decamer observed from the top (**left**) and side (**right**). (**C**) The Rru-EncFtn dimer showing A–A (FOC dimer) interactions. The FOC is indicated with an arrow. (**D**) A close-up view of the Rru-EncFtn FOC coordinating two Fe^+2^ ions as observed from outside the EncFtn (**left**) and from the central channel (**right**). (**E**) The structure of the *E. coli* Bfr monomer (PDB ID: 3e1m). (**F**) A close-up view of the *E. coli* Bfr FOC as observed from inside (**left**) and outside (**right**) of the cage.

FLP-TPs have ~100 amino acids (aa) long structured N-terminal domains and ~10–40 aa long disordered C-terminal domains with conserved TP sequences at their C-termini (Figure 3A). The TP sequences can vary (Table 2). In general, the FLP-TPs have the consensus sequence L(F)X_1_V(I)X_2_X_3_L(I). However, a minimum consensus sequence can be derived from the *T. maritima* encapsulin structure that shows the TP binding site of the FLP (PDB ID: 3dkt), which is LXI(L)X [41]. For most FLP-TPs, X_1_ can be G, T, or S, while X_2_X_3_ can be GS or GT (Table 2).

The N-terminal domain has a 3_10_-helix (H1) preceding the two long α-helices (H2 and H3). The long helices are followed by a shorter α-helix (H4). The N-terminal domain oligomerizes to form a decamer with D5 symmetry (Figure 3B). Each decamer is formed by five repeating units of two antiparallel dimers interacting via the residues on the A and B sides of H2 and H3, forming repeating A–A and B–B interfaces. The H4 extends towards the adjacent dimeric subunit at the outer circumference of the ring and interacts predominantly with the H3 and H4 of that subunit. The decamer has a three-layered annular structure with a diameter of ~7–8 nm and a thickness of ~4.5 nm. Decamer assembly forms a hollow channel called the central channel. The N-termini protrude towards the central channel from both ends. The diameter of the central channel is ~2.5 nm. However, it can narrow down to ~1 nm in some regions due to the protruding N-termini as observed in the *P. furiosus* encapsulated fusion–FLP (Pfc_EncFtn) crystal structure (PDB ID: 5n5e) [28]. The disordered C-terminal domain cannot be seen in any crystal structures. The N-terminal domains of s-FLPs and fusion–FLPs are structurally homologous to FLP-TPs and form decamers (Figure 2). It has been shown that *M. xanthus* encapsulin EncA (T = 3) can encapsulate up to 12 FLP decamers [35]. *P. furiosus* encapsulin is also T = 3 with 180 copies (PDB ID: 2e0z) [44]. Since the fusion–FLP is fused to the N-terminus of the protomer in a T = 3 shell, there will be 180 copies of the fusion–FLP corresponding to 18 decamers, which is unlikely to match with the encapsulin symmetry. Therefore, the encapsulin symmetry, or the fusion–FLP symmetry, may differ from the crystallographic symmetry. The binding of the cargo to the encapsulin can affect its symmetry. For instance, in the absence of cargo proteins, *M. xanthus* encapsulins form both T = 1 and T = 3 particles, but in the presence of the cargo, only T = 3 particles are formed [48]. On the other hand, Ross et al. observed dimeric *H. ochraceum* FLPs in the absence of Fe^+2^, suggesting that FLPs can also adopt different oligomeric states [56]. Interestingly, s-FLPs were initially classified as non-encapsulated FLPs since they lack a TP in their C-termini [57]. However, the crystal structure shows that these FLPs can form decamers similar to the other encapsulated FLPs (PDB ID: 3k6c, unpublished). It is unclear whether the short 10 aa disordered C-terminal region contains an undefined binding sequence. Since fusion–FLPs are connected to the protomer via a short 8 aa peptide, s-FLPs can theoretically be encapsulated without steric hindrance between the cargo and shell proteins (Table 2).

The dimeric subunit of FLP-TPs resembles the four α-helix bundle structures of Ftns and Bfrs (Figure 3C,E). A ferroxidase center (FOC) is formed at the A–A interface (FOC dimer interface) of each dimeric subunit (Figure 3). Similar to Ftns, Bfrs, and diiron-carboxylates, FLP-TPs, fusion–FLPs, and s-FLPs have diiron binding sites consisting of two consecutive helices and a conserved EXXH motif (Figure 2C) [58]. The FOC has a two-fold symmetry axis, and each monomer contributes identical iron coordinating residues. At the FOC, the Fe^+2^ atoms are coordinated by two bridging glutamic acid residues, a histidine and a bidentate glutamic acid residue (Figure 3D,E). In the crystal structures, the FOC Fe^+2^ atoms usually are either five-coordinated (coordination number = 5, CN = 5) or six-coordinated (CN = 6), where the sixth coordinating molecule is either water or a chemical, such as glycolic acid, present in the crystallization condition. FOCs of s-FLPs and fusion–FLPs are also similar to those of FLP-TPs. The B–B interface is called the non–FOC dimer interface and is mediated by a mixture of hydrophobic interactions, hydrogen bonds, and salt bridges [28]. The interactions at the non–FOC dimer interface are more extensive than those at the FOC dimer interface. For example, ΔG is −7.8 kcal/M and −34.1 kcal/M for the A–A and B–B interfaces, respectively, of *H. ochraceum* EncFtn (Hoch_EncFtn, calculated by PISA, from the crystal structure PDB ID: 5n5f [28]). Ross et al. performed an in-depth analysis of the Hoch_EncFtn pentamers of dimers assembly pathway using native mass spectrometry and hydrogen-deuterium exchange mass spectrometry [56]. Their analysis shows that decameric assembly formation is iron dependent and it is associated by the addition of the non–FOC dimers via the formation of the FOC interface upon iron binding.

**Figure 4:**
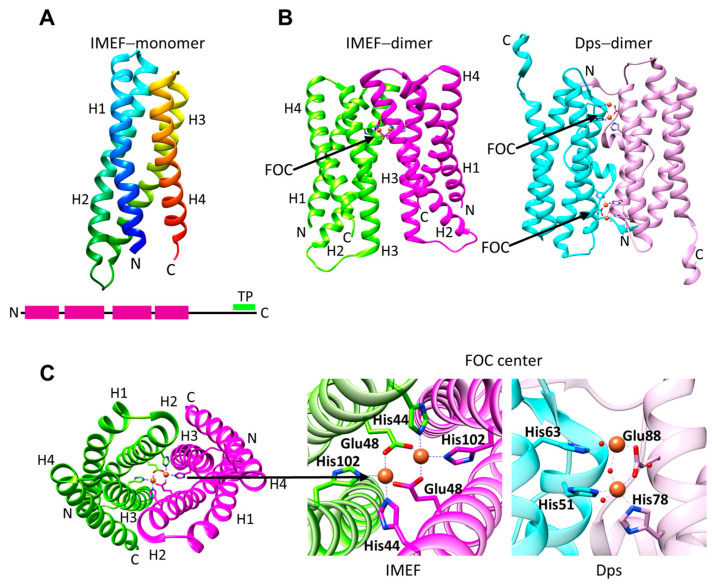
Structure of IMEF. (**A**) The Qt-IMEF monomer (PDB ID: 6n63). The disordered C-terminal region is not visible in the crystal structure. The secondary structure is shown as a scheme at the bottom: α-helices are shown in magenta, and the C-terminal TP is indicated with a green line. (**B**) The Qt-IMEF dimer (**left**) and *N. punctiforme* Dps (**right**). FOCs are indicated with black arrows. (**C**) Qt-IMEF and Dps FOCs. Qt-IMEF FOC as viewed from the top (**left**). Close-up view of Qt-IMEF FOC coordinating two Fe^+2^ ions as observed from the top (**middle**) and close-up view of Np-Dps FOC coordinating two Fe^+2^ ions inside the cage.

Unlike other encapsulated FLPs, IMEF monomers have a four−α-helical bundle structure, and their sequence lacks the conserved EXXH motif (PDB ID: 6n63, Figure 4A,B) [45]. IMEF is more distant from the other FLPs (Figure 2) and resembles Dps proteins [45]. IMEF systems are confined to spore-forming Firmicutes [45]. The monomers form face-to-face parallel dimers, where the FOC is formed by two histidine and two bridging glutamic acid residues located on helices H2 and H3 of the monomers (Figure 4C). In Dps proteins, the FOC site is not symmetric, and Fe^+2^ is coordinated by one or two histidines, a bridging glutamic acid and water molecules [59].

Here, we would like to mention that the nomenclature of the encapsulated FLPs can be confusing since some groups use the general term EncFtn, which stands for encapsulated FLP, and an abbreviation for the organism as a specifier such as Pfc (*P. furiosus*), Rru (*R. rubrum*), or Hoch (*H. ochraceum*) [28,30,55]. For instance, Pfc_EncFtn stands for the *P. furiosus* encapsulated fusion–FLP. Some other groups prefer to use more specific names, such as EncB, for one of the encapsulated FLP-TPs of *M. xanthus* [35]. In yet other cases, simply “FLP” [60] or IMEF [45] is used to define the *T. maritima* (Tm) FLP-TP or *Q. thermotolerance* FLP, respectively. We will use the more general term EncFtn for FLP-TP, fusion–TP, s-FLP, and IMEF for the diverse class of *Q. thermotolerance* FLP homologs.

## 4. Ferroxidase Activity of EncFtns and IMEF

**Figure 5:**
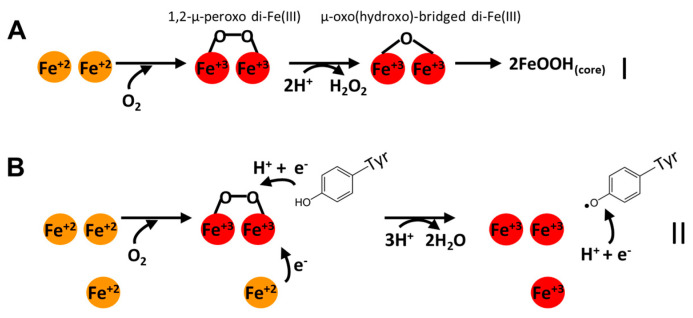
Proposed ferroxidase mechanisms for Ftns and Bfrs. Fe^+2^ ions are shown as orange spheres, and Fe^+3^ ions are shown as red spheres. (**A**) In the first pathway, two Fe^+2^ are oxidized with O_2_, forming a blue intermediate 1,2-μ-peroxo di-Fe(III), which decays to μ-oxo(hydroxo)-bridged di-Fe(III), releasing H_2_O_2_. The μ-oxo(hydroxo)-bridged di-Fe(III) is eventually replaced by the incoming Fe^+2^ ions and transports to the inner cavity to form the mineral core. (**B**) Two Fe^+2^ are oxidized with O_2_ in the second pathway, forming a blue intermediate 1,2-μ-peroxo di-Fe(III). The intermediate receives one e^-^ from a nearby Fe^+2^ and another e^-^ from a nearby tyrosine, forming 3 Fe^+3^, H_2_O, and a tyrosine radical. The tyrosine radical is reduced to tyrosine by receiving one e^−^ from an unknown source.

The ferroxidase mechanisms of EncFtns and IMEF are not known. EncFtn FOCs are structurally similar to both Ftn and Bfr FOCs, but like Bfrs, they have higher symmetry than Ftn FOCs. Therefore, they may adopt similar ferroxidase mechanisms. However, different pathways have been proposed for the ferroxidase activity of Ftns or Bfrs [29,38,61,62,63,64]. Here, we will mention the two widely accepted pathways. In the first pathway, there are two Fe^+2^ ions at the FOC iron binding sites (A site and B site). O_2_ simultaneously oxidizes the two Fe^+2^ ions and reduces O_2_ to H_2_O_2_ [61,63,64]. This reaction proceeds via the formation of the blue intermediate [Fe^+3^-O-O-Fe^+3^]^+2^ (1,2-μ-peroxo di-Fe(III)), which decays to [Fe_2_O-(OH)_2_]^+2^ (μ-oxo(hydroxo)-bridged di-Fe(III)) releasing H_2_O_2_ in stoichiometric amounts. It has been suggested that the μ-oxo diferric complex at the FOC is displaced by the incoming Fe^+2^ ions and transfers from the protein cavity to the iron core for storage in a stable 2FeOOH_(core)_ form (Figure 5A) [64]. If this oxidation step is carried out with H_2_O_2_ instead of O_2_, H_2_O is released instead of H_2_O_2_ during the decay of the blue intermediate, preventing the formation of reactive oxygen species (ROS). The second pathway is similar to the first one except that it involves a third, lower affinity, Fe^+2^ binding site, the C site, and a nearby tyrosine residue. In this pathway, the two FOC sites and the C site are occupied by Fe^+2^ ions. The reaction of the two FOC Fe^+2^ ions with O_2_ forms the blue intermediate [Fe^+3^-O-O-Fe^+3^]^+2^. The third Fe^+2^ at the C site reacts with this intermediate (Figure 5B). The fourth electron for complete reduction of molecular oxygen to H_2_O is proposed to be provided by the conserved tyrosine near the FOC [29]. The second pathway might be more advantageous since the presence of a nearby Fe^+2^ ion as the source of an extra reducing equivalent in combination with a cation-radical-forming nearby tyrosine creates a very efficient mechanism to reduce molecular O_2_ in a single, four-electron step without the formation of ROS [65]. The same FOC Fe^+3^ displacement by the incoming Fe^+2^ ions model is also suggested for this pathway [29].

The ferroxidase activities of several EncFtns, including Rru-EncFtn, Hoch-EncFtn, Pfu-EncFtn, and Tm-EncFtn (UniProt ID: Q9WZP3), have been measured under aerobic conditions [28,30,55,60]. Mutating the Rru-EncFtn FOC iron coordinating residues Glu32, Glu62, and His65 to alanine confirms the involvement of these residues in ferroxidase activity [55]. The bridging Glu62Ala mutation results in a complete loss of ferroxidase activity, while the mutations of the bidentate Glu32Ala and the His65Ala result in ~55–40% reduced ferroxidase activity [55]. The decrease in the ferroxidase activity has been attributed to poor metal coordination in Glu32Ala and His65Ala, and the loss of metal coordination in Glu62Ala mutants. In Rru-EncFtn and Tm-EncFtn, the presence of the encapsulin shell greatly enhances the ferroxidase activity but a mechanism for the involvement of the shell in the ferroxidase activity has not been proposed [55].

IMEF ferroxidase activity under aerobic conditions has also been measured [45]. Interestingly, IMEF without the encapsulin shell shows sigmoidal kinetics of iron oxidation, more characteristic of an autocatalytic mineral surface mechanism in which additional iron oxidation occurs at the surface of the developing iron core [45,66]. On the other hand, the Qt-IMEF encapsulin system shows a typical hyperbolic enzyme curve [45]. Based on these observations, the authors proposed that the encapsulin shell controls the flux of iron to the inside of the compartment leading to a controlled concentration of soluble iron into the encapsulin interior, preventing uncontrolled autocatalytic mineralization which can lead to bulk precipitation of iron inside the encapsulin [45].

## 5. Additional Metal Binding Sites, Fe^+2^ Entry, and Fe^+3^ Exit Routes

Some of the EncFtn crystal structures have been obtained in the presence of calcium. The presence of a second divalent metal in high concentrations (in the range of 100 mM) adds a layer of complication since Ca^+2^ can compete with the much less abundant Fe^+2^ for binding lower affinity metal binding sites. As a result, the crystal structures of some EncFtns show some metal binding sites occupied with Ca^+2^ [30,48,55]. The Ca^+2^ binding sites observed in these crystal structures are likely lower affinity Fe^+2^ binding sites. This assumption is further supported by the observation that Zn^+2^ efficiently inhibits the EncFtn ferroxidase activity suggesting that other divalent metal ions can compete for Fe^+2^ binding sites [28,30]. Also, Ca^+2^ has not been detected in the purified protein samples, confirming that Ca^+2^ binding is not specific [55].

**Figure 6:**
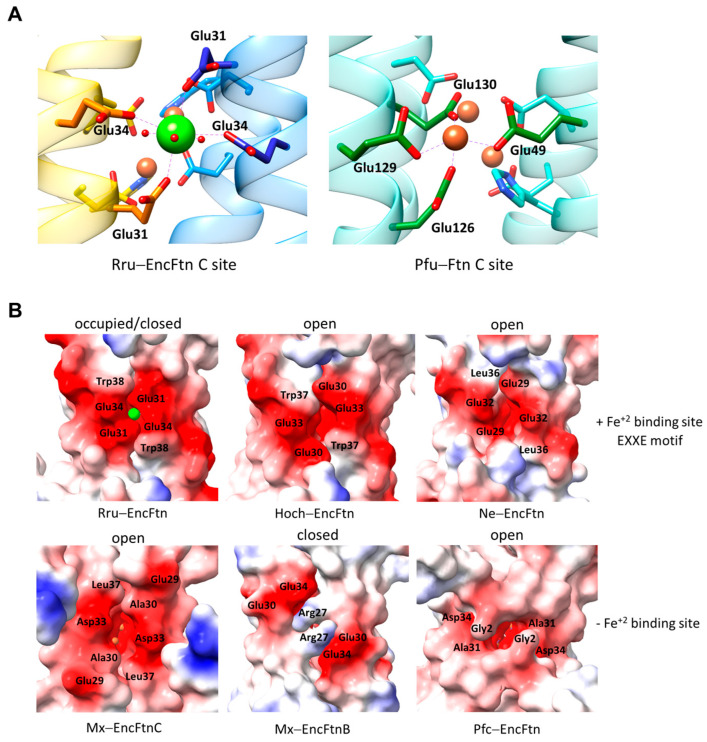
EncFtn C site. (**A**) The proposed EncFtn C site of Rru-EncFtn as observed from the central channel (**left**) and the Pfu-Ftn C site as observed from inside the cage (**right**, PDB ID: 2jd7, [69]). The Ca^+2^ is shown as a green sphere, the Fe^+2^ ions bound to the C site or FOC are shown as brown, and water molecules as red spheres. (**B**) An electrostatic surface potential map of EncFtns shows a conserved gateway to the FOC, as observed from the central channel. The first row shows EncFtns with a conserved EXXE signature motif, and the second row shows the ones without it. The residues forming the gateway or forming the EncFtn C site are indicated.

In crystal structures of EncFtns, additional metal binding sites have been identified. The first site has been observed in the crystal structure of Rru-EncFtn and is formed by four glutamic acid residues, Glu31 and Glu34, from each adjacent H1 and has the signature sequence EXXE (Figure 6A) [55]. In the crystal structure, this site is located on the inner surface of the EncFtn. It is occupied by a CN = 7 Ca^+2^ ion coordinated by four unidentate glutamic acid residues and three water molecules. The Ca^+2^ ion is ~9 Å away from the nearest FOC Fe^+2^. By comparison, the authors decided this site is analogous to site C of the Ftns (Figure 6A). Therefore, we will use the term “EncFtn C site” to define this site [30,55]. Glu31 is not conserved among the EncFtns and can be replaced with alanine or arginine residues. At the same time, Glu34 (or Asp34 in some cases) is highly conserved (Figure 2C).

Rru-EncFtn Glu31 and Glu34 to alanine mutations disrupt the metal coordination site and increase the ferroxidase activity [30]. Zn^+2^ inhibition of ferroxidase activity shows that the EncFtn C site allows the passage of other divalent metals [30]. Based on these observations, the authors concluded that the EncFtn C site has a dual action. First, the electronegative site attracts the positively charged metal ions, and second, it acts as a gateway that controls the entry of the Fe^+2^ influx into the FOC by restricting the free flow of ions to the FOC, thereby regulating the ferroxidase activity. This site is conserved in more closely related Rru-EncFtn, Hoch-EncFtn, and Ne-EncFtn (Figure 2C and Figure 6B). The increase in the ferroxidase activity caused by disrupting the metal coordination ability of the EncFtn C site indicates that this site is not directly involved in an electron transfer function, as suggested in the second pathway of the Bfr/Ftn ferroxidase mechanism. Although the proposed EncFtn C site is not conserved in all the EncFtns, there is a structurally conserved electronegative gateway from the EncFtn central channel to the FOC site, which might allow free diffusion of cations into or out of the FOC (Figure 6B). In Ftns, the role of the C site remains controversial. In some cases, the C site was proposed to act as a transit site of iron from the three-fold entry channel to the FOC by creating an electropotential difference between the FOC and the inner cavity of the Ftn [67]. In other cases, it has been proposed that it plays a role as a gateway in the passage of iron as a transient form to the ferrihydrite stage for final storage [29]. In EncFtns, the polarity and the availability of these sites depend on the surrounding residues and also possibly on the central channel protruding N-termini as observed in the Pfu-EncFtn structure (Figure 6B) [28,30,48,55]. In the Mx-EncFtnB structure, the gateway is blocked by two nearby arginine residues (Arg27) that form pi-pi stacking interactions. However, arginine–arginine interactions are highly susceptible to the polarity of the surrounding environment [68], and this interaction might be due to the crystallization conditions. This Arg27 is not conserved in other structurally characterized EncFtns. Mx-EncFtnB and its homologs form a distinct class of EncFtns; therefore, these proteins might have adopted a unique gateway control mechanism.

**Figure 7:**
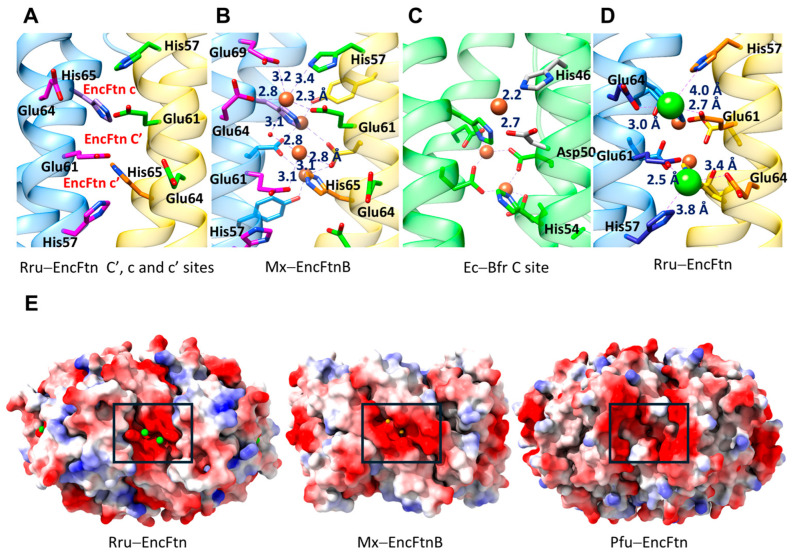
EncFtn C’, c and c’ sites. (**A**) The Rru-EncFtn FOC dimer shows the conserved residues in the HXXXE motif and the FOC histidine, His65, as observed from outside of EncFtn. The proposed EncFtn C’, c, and c’ sites are indicated. (**B**) The Mx-EncFtnB (PDB ID: 7s5c) FOC dimer showing Fe^+2^ coordination by EncFtn C’ and EncFtn c sites. (**C**) The *E. coli* Bfr (Ec-Bfr, PDB ID: *3e1m*) showing Bfr C site Fe^+2^ coordinating residues as observed from inside the cage. (**D**) The Rru-EncFtn (PDB ID: 6suw) FOC dimer showing Ca^+2^ coordination by EncFtn C’ and EncFtn c sites. (**E**) An electrostatic surface potential (ESP) map of the Rru-EncFtn decamer with two Ca^+2^ ions bound to the EncFtn c and EncFtn C’ sites (**left**); ESP map of the Mx-EncFtnB decamer with two Fe^+2^ ions bound to the EncFtn c and EncFtn C’ sites (**middle**); and an ESP map of the Pfu-EncFtn decamer showing similar metal binding sites on its outer surface.

Sequence analysis shows that in the bacterial EncFtns discussed here, a signature HXXXE motif precedes the conserved E_62_X_1_X_2_H_65_ FOC iron binding motif (numbering matches Rru-EncFtn sequence, Figure 2C). In addition, X_2_ in the FOC iron binding motif is a conserved glutamic acid residue (Glu64). Therefore, these bacterial EncFtns have a conserved HXXXE*E_62_*XE*H_65_* motif (the residues represented with italic letters correspond to the FOC iron coordinating residues). Four glutamic acid residues, Glu61 and Glu64, from each adjacent α-helix, form an electronegative pocket on the opposite side of the FOC at the outer circumference of the EncFtn. Although the EXXE motif resembles the Ftn C site, the geometry is distorted due to a rotational shift of the α-helices, increasing the distance between the glutamic acid residues on opposite sites (Figure 7A). Therefore, a single Fe^+2^ atom cannot be coordinated by all four glutamic acid residues. Here, we define this site as the EncFtn C’ site.

It has been shown that Bfrs have a conserved “C site” like Ftns. However, the coordinating residues are a histidine and an aspartic/glutamic acid residue with the signature motif HXXXD/E (Figure 7C) [29,31,70]. The Bfr C site was proposed to play an important role in electron transfer [71,72]. Interestingly, the residues from two adjacent helices form two symmetrical potential metal binding sites at the outer circumference of the EncFtn, which resemble the Bfr C site (Figure 7A,C). These potential sites (EncFtn c site and EncFtn c’ site) are formed by His57 and Glu61 and symmetrically located above and below the EncFtn site C’, ~10 Å away from the FOC. In archaeal fusion–FLPs, the HXXXE*E_62_*XE*H_65_* motif is replaced with DXXXE*E_62_*XA(T)*H_65_*.

**Figure 8:**
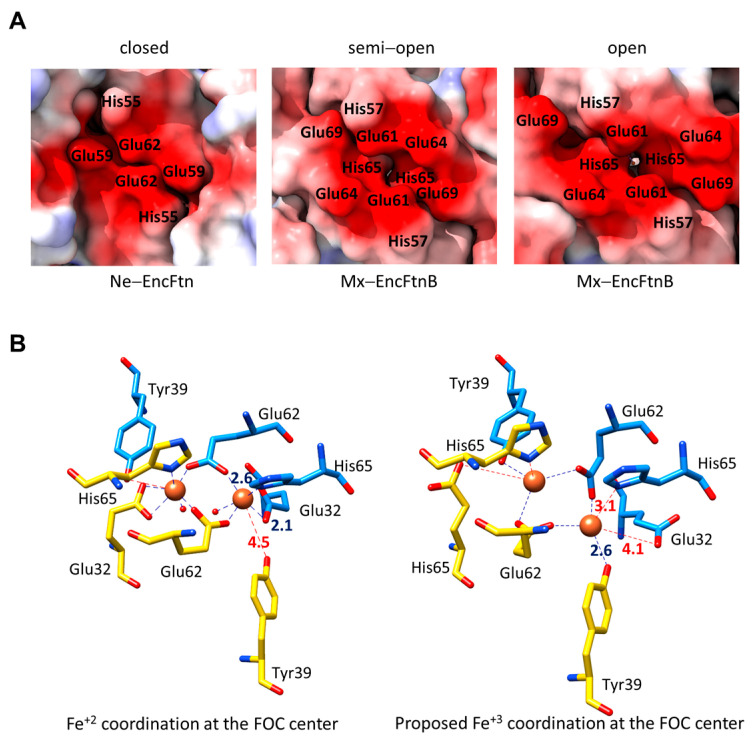
Conformational changes at the EncFtn C’ site and the FOC center. (**A**) A close-up view of ESP maps of the Ne-EncFtn and Mx-EncFtnB FOC dimers showing the “closed”, “semi–open”, and “open” states of the EncFtn C’ site. (**B**) Fe^+2^ coordination by the Rru-EncFtn FOC residues (**left**), and proposed Fe^+3^ coordination by the Mx-EncFtnB FOC residues (**right**).

Multiple crystal structures of Rru-EncFtn and Mx-EncFtnB show loosely bound Ca^+2^ and Fe^+2^ ions in these three consecutive proposed sites (Figure 7B,D) [30,48,55]. The observed metal ions show poor coordination geometry (distance RMSD 0.06 to 0.4 Å) and, in some cases, partial occupancy, suggesting that these are transitory sites. These three consecutive potential metal binding sites form a highly electronegative surface on the outer circumference of the bacterial EncFtns proximal to the FOC (Figure 7E). Unlike the EncFtn C site, the EncFtn C’ site does not show a generally “open” state that provides access to the FOC. However, in different EncFtn structures, this site can be observed in closed, semi-open, and open states, which are determined by the conformation of the FOC His65 residues and the outer surface conserved histidine and glutamic acid residues (Figure 8A) [30,48]. For *P. aeruginosa* BfrB, it has been proposed that the FOC histidine, His130, goes under side-chain rearrangement to switch between “gate open” and “gate closed” conformations to allow Fe^+3^ displacement by Fe^+2^ [31]. In fusion–FLPs, EncFtn c and EncFtn c’ like sites are also formed by the conserved Asp57, Glu61, and a conserved glutamic acid residue, Glu68 (Figure 7E).

Based on sequence conservation, proximity to the FOC histidine residues, and high electronegative potential, we proposed the EncFtn C’ site as the Fe^+2^ entry site to the FOC as an alternative to the EncFtn C site proposed by He et al. (2019) [48]. Based on this, the outer surface electronegative pocket can act as a metal attractor, and the Fe^+2^ ions bound to the surface via EncFtn c or c’ sites diffuse through the EncFtn C’ site towards the FOC upon oxidation of Fe^+2^ to Fe^+3^. Diffusion through the EncFtn C’ site is likely controlled by the movement of His65 away from the oxidation site, releasing the Fe^+3^. This theory is supported by the observation of conformational changes at the FOC of Mx-EncFtnB, where Glu32 residues no longer coordinate the CN = 4 Fe ions and His65 residues are moving away from the Fe ions (Figure 7D) [48]. Meanwhile, Fe ions, likely Fe^+3^, are coordinated by the two bridging Glu62 and Tyr39. The formation of an intermediate Fe(III)-tyrosinate has been observed in the H subunit of ferritin which was proposed to increase the rate of biomineralization [73]. The Tyr39 is highly conserved in EncFtns and is located at a distance of ~4.5 Å from the Fe^+2^ ions coordinated at the FOC (Figure 7D). Tyrosine residues near the FOCs are also conserved in Ftns and Bfr, and several studies highlight the importance of that residue for the ferroxidase activity [29,65,74,75,76]. In Bfrs, this conserved tyrosine residue has been proposed to function in electron transfer from the inner surface site to the FOC by forming a transient radical (Figure 5B) [29,65,74,75,76]. The role of this tyrosine remains to be elucidated for the EncFtns, which might shed some light on understanding the ferroxidase mechanisms of EncFtns.

On the other hand, the conserved electronegative gateway (and the EncFtn C site, if present) located at the inner surface of the EncFtn can act as the Fe^+3^ exit site where the surrounding environment can control the diffusion rate. He et al. estimated that an encapsulated EncFtn can bind up to 48 iron ions using mass spectrometry (MS) analysis, which confirms the presence of secondary metal binding sites on the inner and outer surface of the EncFtns [55]. However, mutational and functional studies are required to elucidate the roles of these potential metal binding sites in the ferroxidase activity of the EncFtns.

## 6. Iron Storage

Ferroxidase activity measurements with EncFtns show that although these proteins have ferroxidase activity without the encapsulin, they do not store iron in the mineralized form [28,45,55,60]. In these assays, Fe^+2^ is oxidized to Fe^+3^, which forms yellow- to red-colored precipitates [55]. It has also been observed that the ferroxidase activity can be significantly enhanced in the presence of the encapsulin shell [55]. TEM images of stained and unstained encapsulins show the presence of dense iron minerals inside the encapsulins [35,45,55]. The *M. xanthus* encapsulin (Mx-Enc) is 32 nm across with a 26 nm internal diameter [35]. The cryo-EM structure of the Mx-Enc shows a 24 nm dense core within the particles, which is rich in iron and phosphorus [35]. Inductively coupled plasma mass spectrometry (ICP-MS) experiments have shown an Fe:P_i_ ratio of 4:1 for the stored iron compared to the 9:1 ratio observed in ferritin [35]. Dark-field STEM experiments revealed that the dense core of the Mx-Enc contains an average of 30,000 Fe atoms. The iron mineral cores appear granular [35].

For Rru-EncFtn, the number of Fe atoms was calculated from iron storage assays, which showed an average of 4100 iron atoms per encapsulin [55]. However, since the authors did not reach the apoferritin control’s maximum iron loading capacity in their assays, they suggested that the capacity of the Rru-EncFtn encapsulin system could be much higher [55]. The iron loading capacity of the Qt-IMEF encapsulin system was determined by electron energy loss spectroscopy (EELS) and estimated to be around 23,000 Fe atoms per particle, corresponding to a 23.6 nm core [45]. These iron cores appeared amorphous. The authors suggested that an amorphous iron form can be more readily mobilized under iron-limited conditions than a crystallized iron mineral [45]. The authors estimated the theoretical size limit imposed by the T = 4 encapsulin protein shell as 36 nm across and extrapolated the highest density observed (3.40 Fe atoms/nm^3^) to the maximum theoretical particle diameter, calculating a maximum number of 83,000 Fe atoms that can potentially be stored by the Qt-IMEF encapsulin system [45]. Here, we note that the core Fe:P_i_ ratio in the Qt-IMEF encapsulin system was estimated as 1:1, which differs from that of the *M. xanthus*-EncFtn encapsulin system. For *E. coli* Ftn, it has been observed that the core iron mineral can take different forms depending on the phosphate, and the absence of phosphate results in the appearance of granular particles, which can explain the observation of amorphous minerals in the Qt-IMEF encapsulin system versus granular minerals in the *M. xanthus*-EncFtn encapsulin system [67].

**Figure 9:**
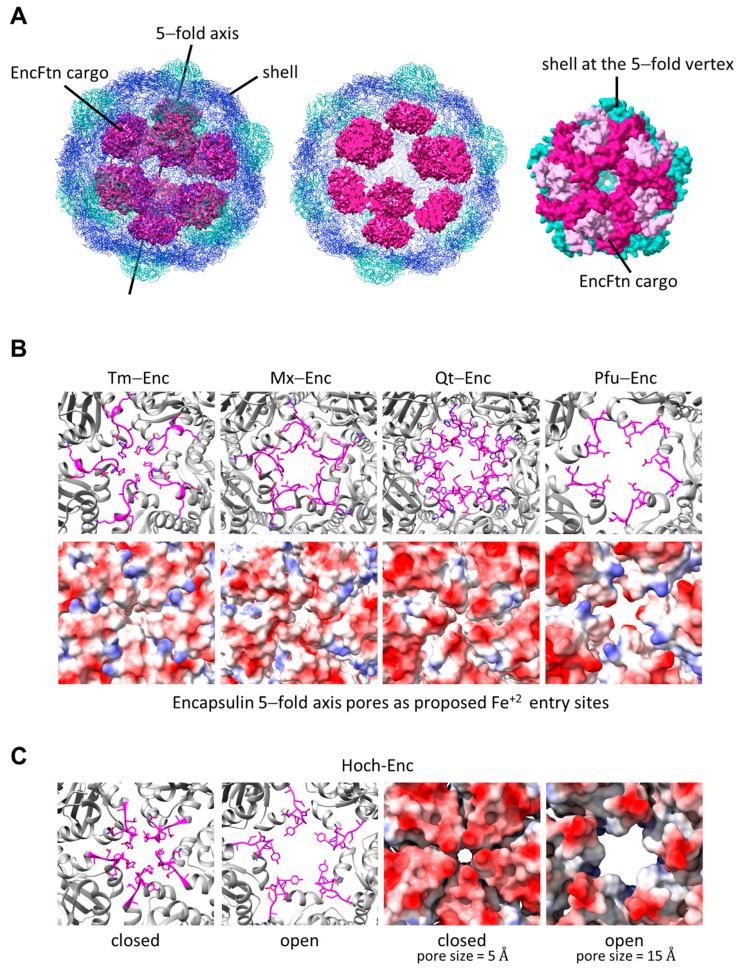
Fe^+2^ entry into the encapsulin from the five-fold axis pores. (**A**) Mx-EncFtnC cargo encapsulated by the *M. xanthus* encapsulin EncA (PDB ID: 7s4q) (**left**). Central slice of encapsulated Mx-EncFtnC structure (**middle**). Mx-EncFtnC decamer and the five-fold vertex shell proteins aligning the five-fold axis pore of the shell with the central channel of the EncFtn, as observed from the central cavity of the encapsulin (**right**). (**B**) Close-up views of five-fold axis pores of encapsulins encapsulating EncFtns (**top**), and the corresponding EPS maps (**bottom**) (Tm-Enc, PDB ID: 7k5w; Mx-Enc, PDB ID: 7s4q; Qt-Enc, PDB ID: 6nj8; and Pfu-Enc, PDB ID: 2e0z). (**C**) Hoch-Enc five-fold axis pores in the “closed” and “open” states (**left**), and the corresponding EPS maps (**right**) (PDB ID: 7oe2 and 7oeu). In EPS maps, electronegative surfaces are shown in red, electropositive surfaces are shown in blue, and neutral areas are shown in white.

Encapsulins have pores that penetrate the shell. These pores are located at the five-, three- and two-fold axes [77]. The pores have distinct local environments with specific positive and negative charge distribution on their inner and outer surfaces. These pores are thought to act as selective channels. Since the EncFtns and IMEF are located within the encapsulin shells, it has been suggested that the Fe^+2^ enters the encapsulin through the negatively charged pores located at the five-fold axes (T = 1 and T = 3) or through pores located at the three-fold and five-fold axes (T = 4) [45,48,60,78,79]. Cryo-EM structures of Tm-EncFtn and Mx-EncFtn encapsulin systems show that the EncFtn cargo is located around the pentameric vertex aligning with the five-fold axis pores (Figure 9A) [48,60,80]. On the other hand, the Hoch-EncFtn encapsulin system shows an asymmetric loading of the EncFtn cargo with tetrahedral symmetry within the encapsulin shell [78]. In that arrangement, only two EncFtn decamers align with the five-fold axes, while the other two are located between the five- and three-fold axes. IMEF TPs bind to the two-fold symmetrical hexameric capsomers, with 42 copies of IMEF dimers per encapsulin [45].

Residues surrounding the five-fold axis pores of iron storage encapsulins are not conserved (Figure 9B). However, the pore-lining residues, tyrosine, asparagine, threonine, or glutamic acid, create an electronegative environment around the pores, which likely attracts the positively charged Fe^+2^ ions (Figure 9B). It has been shown that five-fold axis pores are dynamic and can differ by up to 10 Å in diameter in their “closed” and “open” states (Figure 9C) [78]. The largest pore diameter of 15 Å has been observed in Hoch-Enc. It is not known whether iron diffuses through the shell pores in free ion form or if it is complexed with low molecular weight thiols or cysteine. However, GSH is ~14 Å, and Fe^+2^ is 0.76 Å, making it possible for a (H_2_O)_5_Fe(II)-GSH complex to diffuse through the 15 Å pores. It has been shown that the natural substrate Fe^+2^ species that transits through the entry channels at the three-fold pores of *Rana catesbeiana* Ftn is [Fe(H_2_O)_6_]^+2^, which is the more likely form considering the pore sizes of ~5–10 Å observed in Mx-Enc, Tm-Enc, and Qt-Enc (Figure 9B) [81].

Since the majority of the organisms having FLP encapsulin systems also encode Bfr, Ftn, or Dps in their genomes, it has been suggested that the primary role of these systems might not be iron storage but rather combating oxidative stress by temporarily storing excess reactive iron under certain conditions [49]. FLP encapsulin systems have similar (in the case of T = 1) or much higher (in the case of T = 3 or T = 4) iron storage capabilities in comparison to Bfr, Ftn, and Dps which might suggest that they function under more extreme conditions [35,45,55]. To our knowledge, there have been no studies that involve a direct comparison of functionalities of Bfr/Ftn/Dps and EncFtns in the same organism under different environmental conditions. However, it has been shown that in *M. tuberculosis*, which has both a Ftn (BfrB) and a Bfr (BfrA), these proteins play different roles depending on the iron concentration in the environment, and their expression is regulated by iron [82]. It was proposed that BfrB, which has higher iron storage capabilities, acts as the preferred iron storage protein under high iron concentrations. On the other hand, BfrA can mediate iron homeostasis under limited iron conditions due to its more efficient iron release capabilities. The presence of two different FLPs in the *M. xanthus* encapsulin system also suggests that some organisms might have evolved to have multiple iron homeostasis proteins to survive under variable conditions.

## 7. Iron Release

Since EncFtns and IMEF are involved in iron storage, an iron release mechanism is also expected to be carried out under iron-limiting conditions. However, electron transfer components are required to reduce mineral core iron to Fe^+2^. Although the iron storage capabilities of encapsulin EncFtn/IMEF systems have been studied, iron release from the encapsulin systems has yet to be shown. While it has been shown that both Ftns and Bfrs can remobilize iron when required [29,31], the mechanism of Fe^+2^ recovery from Ftns needs to be clarified. It has been proposed for the eukaryotic ferritins that the core Fe^+3^ could be reduced to Fe^+2^ by a small chemical such as dihydroflavin or a flavoprotein [29]. However, this might require partial unfolding of the pores or complete ferritin degradation to provide access to the mineral core [29]. The iron release for Bfrs has been better characterized [29,31]. *Pseudomonas aeruginosa* (*P. aeruginosa*) bacterioferritin PaBfr interacts with bacterioferritin-associated ferredoxin (Bfd) and a long-distance (~18 Å) electron transfer through the heme group of the Bfr results in the reduction of Fe^+3^ to Fe^+2^ [31].

**Figure 10:**
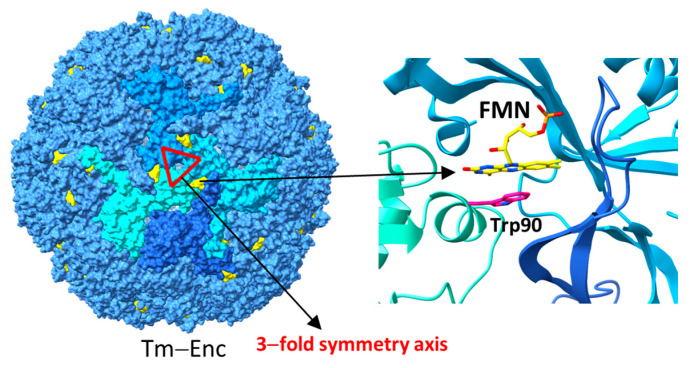
The FMN binding site on Tm-EncT (PDB ID: 7kq5) (**left**) and a close-up view of the FMN binding site highlighting the conserved tryptophan residue coordinating the FMN (**right**).

It has been shown that there is a flavin binding site on the *T. maritima* encapsulin shell which involves a conserved tryptophan residue, W90 (Figure 10) [47,60]. The flavin-binding site consists of residues from three different subunits, distinct from the trimer that forms the three-fold axis, and it can bind both flavin mononucleotide (FMN) and riboflavin [60]. Ferroxidase assays with the WT *T. maritima* and a flavin-binding deficient W90E mutant showed no difference in iron oxidation activity [60]. This is not surprising since other ferroxidases and IMEF also show ferroxidase activity without flavin [30,45,55]. The authors also considered the possibility of this flavin to reduce Fe^+3^ in order to remobilize iron under iron-limiting conditions, but this could not be shown by in vitro experiments [60]. However, an unidentified molecule or protein required as a co-factor or electron carrier might be missing from these in vitro experiments. In line with this, many IMEF operons encode a conserved 2Fe–2S ferredoxin, homologous to Bfr-associated ferredoxins, which carries an N-terminal motif similar to the conserved C-terminal TPs found in confirmed Family 1 cargo proteins and was shown to co-purify with the T = 4 shell under heterologous expression conditions [45,49]. It is not clear if this ferredoxin is encapsulated or not. However, ferredoxins or flavoproteins may act through interactions with the encapsulin shells and facilitate long-distance electron transfers through aromatic amino acid networks.

## 8. Implications of Unusual EncFtn and Encapsulin Systems

**Figure 11:**
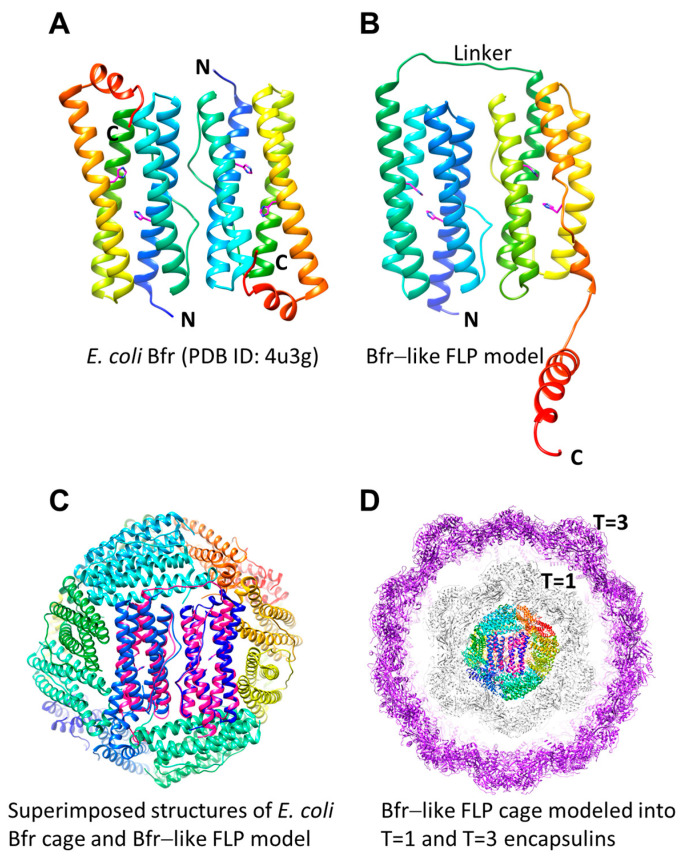
Model of a Bfr-like FLP. (**A**) Two antiparallel four-helix-bundle monomers as arranged in one face of the *E. coli* bacterioferritin cage (PDB ID: 4u3g) colored from N-terminus (blue) to C-terminus (red) and with the two histidine residues at each of the two Bfr FOCs indicated (magenta). (**B**) The AlphaFold predicted structure of the Bfr-like FLP (EDP73503.1) depicted as in (**A**). (**C**) The Bfr-like FLP (magenta) aligned to the *E. coli* Bfr cage (PDB ID: 4u3g) as in (**C**). (**D**) The Bfr-like FLP as in (**C**) centered within a T = 1 encapsulin cage (PDB ID: 7s21, grey) and a T = 3 encapsulin cage (PDB ID: 7s20, dark purple).

In addition to EncFtns and IMEF, putative FLPs that are structurally homologous to hemerythrin, and Bfr with C-terminal TPs have been identified [53,57]. The putative Bfr-like FLP cargo proteins (previously named Flp + Flp, [57]) are of particular interest due to the capability of Bfr-like proteins to form cages. AlphaFold models (available from the AlphaFold protein structure database, https://alphafold.ebi.ac.uk/, accessed on 1 April 2024) of putative encapsulated Bfr-like FLPs show two consecutive four α-helical bundles connected with a linker and a long, disordered C-terminal domain. In general, these Bfr-like FLPs have only one conserved symmetrical EncFtn diiron binding FOC located in the first α-helical bundle, while the second α-helical bundle has variable centers. The double α-helical bundles resemble a Bfr homodimer (PDB ID: 4u3g, [83] Figure 11A,B). Interestingly, superimposed structures of the *E. coli* Bfr cage and the putative Bfr-like FLP model show that the Bfr-like FLP can form a similar 12-mer homooligomeric cage which can theoretically be encapsulated by a T = 1 or a T = 3 cage (Figure 11C,D). It has been proposed that *Mycobacterium tuberculosis* has a Ftn-like four α-helical bundle cargo-protein (Mt-BfrB, named BfrB since the authors initially thought it was a Bfr homolog) which can be encapsulated and has ferroxidase activity within the encapsulin [34]. Although a cryo-EM structure is not available, negative stain images show cage-like structures within the T = 1 encapsulin [34]. Later on, the authors deposited an unpublished crystal structure of Mt-BfrB (PDB ID: 3uno) which shows a typical 24-mer Ftn cage.

Although the physiological advantage of a “cage in a cage” structure is not immediately available to us, in theory it might serve as a means of compartmentalization of two different metabolic processes. We hope that future structural and functional studies will shed some light on this concept.

## 9. Summary

**Figure 12:**
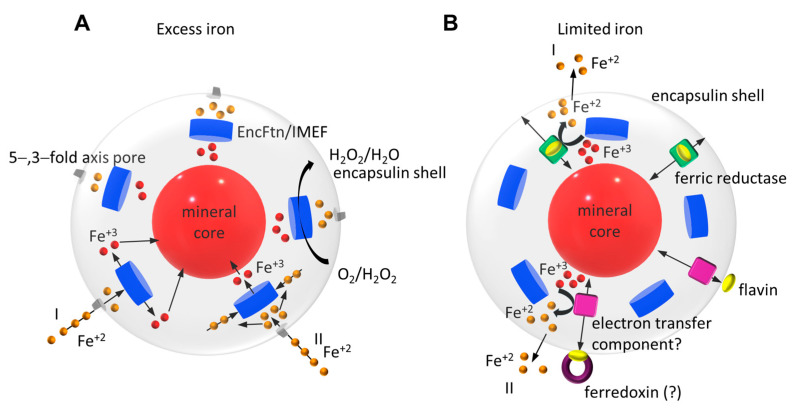
Proposed general models for the iron storage (**A**) and release (**B**) functions of encapsulated FLPs. In iron storage, Fe^+2^ enters the encapsulin from the negatively charged five-fold axis pores (EncFtn) or both the five-and three-fold axis pores (IMEF) by diffusion. Fe^+2^ then enters the FOC either through the EncFtn C site/gateway from the central channel (I) or through the EncFtn C’ site from the outer surface (II). At the FOC, Fe^+2^ is oxidized to Fe^+3^ by utilizing either O_2_ or H_2_O_2_. Fe^+3^ is then displaced by the incoming Fe^+2^ and released to the central cavity of the encapsulin via the EncFtn C’ site from the outer surface (I) or the EncFtn C site/gateway (II) and forms an iron-phosphate mineral core. In iron release, an encapsulated ferric reductase or a ferredoxin that can reach the mineral core reduces Fe^+3^ to Fe^+2^ by the redox reaction of bound flavin (FMN or riboflavin) under iron–limited conditions (I). If the flavin is bound to the encapsulin shell, a cytoplasmic ferredoxin or similar protein can interact with the shell and reduce flavin. Then, the electrons can be transported to the mineral core by an unidentified electron carrier partner (II).

EncFtns, IMEFs, and their corresponding encapsulins play a significant role in iron storage in mineral form by oxidation of Fe^+2^ to Fe^+3^ (Figure 12A). Since H_2_O_2_ can also be used as an oxidizing agent during the ferroxidase activity, the FLP-Enc systems can protect the organism against oxidative stress. In line with this, it has been observed that the expression of *T. maritima* and *M. xanthus* encapsulins is upregulated during oxidative stress [35,84]. In addition, in *M. xanthus,* deletion of the encapsulin shell EncA gene makes *M. xanthus* highly susceptible to hydrogen peroxide-induced oxidative stress and results in phenotypic growth defects [35,85]. Since the majority of spore-forming Firmicutes lack other iron storage systems, such as Ftns and Bfrs, the Qt-IMEF encapsulin system has been suggested to act as the primary iron homeostasis system [45]. Other EncFtn encapsulin systems, such as the Mx-EncFtn and Tm-EncFtn encapsulin systems, have been suggested to act as secondary iron storage systems induced by oxidative stress [35,84]. Although some encapsulated FLPs have been characterized, limited information is available for iron entry, ferroxidase, and iron storage mechanisms. Future studies are required to understand these mechanisms better. Finally, we recently identified a novel bacterial family of NADH/NADPH-dependent flavin-binding ferric reductases with conserved C-termini TPs (EncD family proteins, where some members were mistakenly assigned as ferroxidases). *M. xanthus* EncD can be encapsulated, reduce the mineral iron core, and release Fe^+2^ from Mx-Enc, confirming that encapsulins can use a ferroxidase-independent mechanism for iron-releasing action (Figure 12B) [86].

## 10. Discussion

Encapsulins are members of a large family of protein-based nanocages with diverse metabolic functions. They have been identified in aerobic and anaerobic bacteria and bacterial and archaeal extremophiles [53]. Although several Family 1 and Family 2 encapsulins have been structurally and functionally characterized, large-scale computational analyses reveal over 6000 encapsulin-like systems in bacterial and archaeal phyla [53]. Family 1 and 2 encapsulins have been identified in antibiotic-resistant ESKAPE pathogens, including *E. coli*, *Klebsiella pneumoniae*, *Acinetobacter baumannii*, and other pathogens [57]. A dye-decolorizing peroxidase (DyP) encapsulin directly involved in oxidative stress during *Mycobacterium tuberculosis* infection shows that encapsulins can play a role in bacterial pathogenesis [87].

Apart from their physiological functions, encapsulins are valuable instruments for nanotechnological applications. Encapsulins from bacteria and extremophiles can withstand a wide pH and temperature range, and they can be engineered to contain surface-exposed shell-fusion proteins. In addition, their cargo can be exchanged with non-native cargo engineered to contain the TPs. Tm-Enc has been used as a nanoreactor to generate blue-light-inducible production of ROS or singlet oxygen, a reactive and short-lived excited state of oxygen, to exert a light-activated phototoxic effect on cancer cells [88,89,90]. In addition, encapsulins have been engineered to serve as effective platforms for targeted drug delivery and nano-vaccine production. For instance, fusion of the hepatocellular carcinoma cell targeting peptide SP94 to Tm-Enc that was crosslinked with the acid-sensitive prodrug aldoxorubicin resulted in the internalization of these encapsulins in HepG2 tumor cells [91]. The cell viability was reduced due to the doxorubicin release in the acidic environment of the tumor cells. An encapsulin-based SARS-CoV-2 vaccine immunogen was generated recently [92]. The Mx-Enc shell protein was used as a scaffold for multivalent display of the monomeric receptor-binding domain derivative (mRBD), which induced high titers of neutralizing antibodies in mice. Encapsulin engineering also proved to be helpful in biological imaging. Mx-Enc encapsulating the EncFtn-fused *Bacillus megatherium* tyrosinase showed melanin production in human cells, generating strong contrast in photoacoustic images [93].

Due to their mineral storage capabilities, EncFtn and encapsulin systems have more specific nanotechnological applications. Human cells expressing the native Mx-EncFtn encapsulin system produced intense signals in MRI analysis [94]. The Mx-EncFtn encapsulin system was also used to generate magnetic nanoparticles for magnetic hyperthermia therapy, where encapsulin-produced magnetic iron oxide nanoparticles efficiently absorb magnetic energy, resulting in a pronounced temperature increase to induce tumor cell toxicity and apoptosis [95]. The Tm-EncFtn encapsulin system was engineered to synthesize size-constrained silver nanoparticles as bactericidal and bacteriostatic agents [89].

Structural and functional analysis of EncFtn, IMEF, and encapsulin systems points to diverse strategies developed by bacteria, archaea, and extremophiles to adapt to their environmental conditions. Large-scale computational analysis of genomic sequences using the conserved TP sequences suggests that the encapsulated FLPs are more diverse than the already known family members [53,57]. In these studies, the authors identified 528 uncharacterized FLPs. In addition to ferroxidases, 2Fe-2S ferredoxins with TP-like TVGSL motifs have been identified in IMEF operons [53]. This analysis highlights the vast number of encapsulin systems that must exist in the biosphere, but also how little they have so far been experimentally characterized, and just how much is potentially yet to be discovered. Although research in encapsulin systems is relatively new, it has already produced several promising biotechnological applications. Continued work in this area and understanding of the mechanistic details of encapsulin systems will provide the knowledge needed to construct more efficient and better controlled systems. Finally, with the characterization of new encapsulin systems and their cargo we will better comprehend the range of possibilities offered by the encapsulin systems.

## Figures and Tables

**Table 1:** Summary of FLPs with X-ray crystal structures deposited in PDB.

PDB ID	Name	UniProt Reference	Organism	Type	Form	Encapsulin T-Number	Organism Type	Amino Acids
5da5	Rru-EncFtn, Rru_A0973	Q2RVS1	*Rhodospirillum rubrum*	FLP-TP	Fe-bound	1 ^1^	Bacteria, anaerobic, photosynthetic	140
3k6c	Ne-EncFtn * NE0167	Q82XT5	*Nitrosomonas europaea*	s-FLP	apo	? ^4^	Bacteria, anaerobic, nitrifying	95
5n5e	Pfc-EncFtn PFC_05175	Q8U1L4	*Pyrococcus* *furiosus*	fusion–FLP	Fe-bound	3 (? ^4^)	Archaea, anaerobic, hyperthermophile	345 ^2^ 1–109 ^2^
5n5f	Hoch-EncFtn Hoch_3836	D0LZ73	*Haliangium ochraceum*	FLP-TP	apo	1	Bacteria, aerobic, myxobacteria	131
7s5c, 7s5k	Mx-EncFtnB * EncB, MXAN_3557	Q1D6H3	*Myxococcus* *xanthus*	FLP-TP	Fe-bound	3	Bacteria, aerobic, myxobacteria	158 ^3^
7s8t	Mx-EncFtnC * EncC, MXAN_4464	Q1D3Y8	*Myxococcus* *xanthus*	FLP-TP	Fe-bound	3	Bacteria, aerobic, myxobacteria	130
6n63	Qt-IMEF * Qs IMEF, Qs IMEF cargo, QY95_01593	A0A0F5HNH9	*Quasibacillus thermotolerans*	IMEF	Fe-bound	4	Bacteria, thermophilic, firmicute, facultative anaerobe	192

^1^ The encapsulin structure is not solved. T = 1 symmetry is deduced from the particle size determined from the negative stain images. ^2^ The encapsulated FLP domain is formed by the aa 1–109, and the encapsulin shell protein (protomer) is formed by the aa 110–345. ^3^ The aa number in the UniProt entry differs from the experimentally determined aa number [35]. ^4^ The question mark (?) is used to define unknown or unconfirmed T-numbers. * These names are different from the published names for these FLPs and are suggested here to simplify the nomenclature of these proteins.

**Table 2:** Sequences of TPs observed in structurally characterized FLPs and homologs.

FLP	Conserved C–Terminal TP Sequences
FLP-TP	
*R. rubrum*	SLGIGSLK(R)
*H.* *ochraceum*	SLGIGSLK
*M. xanthus* (**EncC**)	RLTVGS(N)L(M)
*M. xanthus* (**EncB**)	PLTV(I)GS(T)L
*T. maritima*	GG(S)D(S)LG(D)I(L)R(G)K(S)L
Fusion–FLP	RSLLKHLP ^1^
s-FLP	LFTD(E/N)KPIAHE ^2^
IMEF	G(S/T)F(L)TVGSLI(L)

^1^ This is not a TP but the fusion peptide between the FLP and the protomer. ^2^ This TP is putative and has not been characterized. The conserved hydrophobic residues involved in TP binding are shown in bold letters.
